# Supplementary material for: Are carbon emissions trading and green financial instruments synergistic? -Comprehensive quantitative research based on content analysis
Source: PLoS One. 2024 Mar 7;19(3):e0298601. doi: 10.1371/journal.pone.0298601 (PMC10919601; doi:10.1371/journal.pone.0298601)
Supplement: S1 Data — (ZIP) [file pone.0298601.s001.zip › Data/地级市Z_F工作报告.zip!/║╙─╧/╞╜╢Ñ╔╜╩╨/└┤╫╘╣1⁄2╓┌║┼í╛┬φ┐╦╩2╛▌═°í┐_2017.docx]

各位代表:

现在,我代表市人民政府,向大会报告工作,请予审议,并请各位政协委员和其他列席人士提出意见。

一、2016年工作回顾

过去的一年，在党中央国务院、省委省政府和市委的坚强领导下，全市上下以新发展理念为引领，坚持稳中求进工作总基调，主动适应经济发展新常态，积极推进供给侧结构性改革，统筹稳增长、促改革、调结构、惠民生、防风险，全市经济稳中有升、社会大局和谐稳定，实现了“十三五”良好开局。

（一）着力稳增长保态势，经济实现平稳增长。狠抓稳企业、扩需求、优环境等措施的落实，力促经济运行稳中向好。初步核算,全市生产总值完成1824.6亿元、增长7.7%，一般公共预算收入124.5亿元、增长5.1%，规模以上工业增加值增长7.6%。企业服务持续深化。坚持每季度召开与重点企业联席会议和现场办公制度，举办53场产销、银企、用工、产学研对接活动，帮助企业解决问题200多个。金融机构新增贷款184.2亿元。4家企业在新三板上市。储备工业用地9189亩，获批建设用地9136.5亩。有效需求持续扩大。实施十大扩消费行动，社会消费品零售总额增长11.8%。深入实施模拟审批，开展集中开工和观摩活动，己二酸己内酰胺一期、极智联合固网智能终端一期等项目建成，郑万铁路、沙河复航平顶山段等项目进展顺利，重点项目完成投资680亿元，带动固定资产投资增长8.1%。争取到中央补助资金4.3亿元、专项建设基金9.2亿元。发展环境持续优化。出台降低企业成本49条措施，制定促进非公有制经济发展优惠政策，民营企业投资信心不断增强。深入开展懒政怠政为官不为问责年活动,发展环境有新的改善。

（二）强力推进结构调整，转型升级步伐加快。出台供给侧结构性改革总体方案和专项方案，加大产业产品结构调整力度。化解过剩产能任务圆满完成。落实职工安置、债务处置、奖励补助等政策，退出煤炭产能503万吨，超额完成年度任务。出台加快消化库存商品房促进房地产业平稳健康发展的意见，实行购房补贴政策，市区商品房去化周期较年初下降19个月。工业结构进一步优化。装备制造业和高新技术产业比重分别提高1.5个和2.3个百分点，能源原材料产业和六大高耗能行业比重分别下降1.1个和0.8个百分点，电气机械制造业增长14.7%，尼龙产业增长20%以上。定期组织产业集聚区观摩，推动与行政区域管理套合，集聚区完成投资563.1亿元。第三产业快速发展。服务业“两区”完成投资54.5亿元，新华怡购城、中原玉石城、盛润广场、万达广场、义乌商贸城等项目加快建设，电商物联网产业园等项目开工。9家金融机构入驻我市，叶县农商行挂牌开业。香山寺创建为国家4A级景区。第三产业增长9.3%，比重提高1.2个百分点。创新能力持续提升。推动建立平煤神马集团上海研究院、平高集团清华大学研究院、生态循环农业上海交大工作站，高新区创建为省首批区域创新创业基地。组织制定国家标准6项。新增国家高新技术企业11家、省级工程技术研究中心6家、省级工程实验室5家。获省科技进步一等奖3项、二等奖2项、三等奖9项，获奖数量和质量居全省前列。企业与高校及科研院所合作开展科研项目134个，其中煤电一体化项目得到李克强总理的肯定。生态建设成效显著。重拳治理大气污染，强力推进“六控”，PM2.5、PM10平均浓度和优良天数完成省定目标。加强水污染防治、水资源管理和水生态保护，地表水断面综合达标率94.1%，饮用水源地水质达标率保持100%。编制完成土壤污染防治实施方案，重金属污染物排放实现零增长。新增造林面积16.4万亩，白龟湖国家湿地公园通过验收。加强能源消耗总量和强度控制，成为全国首批工业资源综合利用示范基地，单位生产总值能耗下降4.8%。

（三）全面深化改革开放，发展动能不断增强。坚持改革开放双轮驱动，加快动能转换。各项改革实现新突破。强力推进驻平央企、省属国企剥离企业办社会职能，与4家省属企业签订“三供一业”分离移交协议，供水、供电、供暖（气）维修改造正加快推进。翔隆不锈钢、洁石焦化混合所有制改革后正式投产，省国控公司向圣光集团债转股工作进展顺利。全面取消和调整非行政许可审批事项，清理规范53项行政审批中介服务事项，编制完成市县两级政府权力、责任和效率清单。“营改增”全面展开，涉企资金基金化改革稳步推进。推广政府与社会资本合作（PPP）模式，147个项目进入市意向库，45个进入省意向库。深化商事制度改革，新登记各类市场主体3.5万户、增长19.7%。完成市级党政机关公车改革。不动产统一登记、价格、电力、供销、盐业等改革稳步推进。对外开放取得新成果。在长三角、珠三角、京津冀、闽东南和东南亚地区举办系列招商活动，成功举办第六届“华合论坛”，共签约项目226个、金额2156亿元。与泰国正大集团、华耀城集团、绿地控股集团、河南投资集团签订战略合作协议。中德生态环保合作试点项目进展顺利。郑州海关和河南出入境检验检疫局平顶山办事处获批。

（四）统筹推进城乡建设，城乡一体协调发展。不断完善推进新型城镇化的体制机制，进一步提升城市规划、建设和管理水平，城镇化率达50.8%、提高1.6个百分点。城市总体规划获国家正式批准，高铁站周边控制性详规、城市设计和路网规划持续完善。实施打通断头路三年行动计划，开发一路北延、新南环路东延等项目加快建设。孟平铁路复线、三洋铁路平顶山段、平宝路改线、平郏快速通道建成通车，鹰城广场改造、中兴路改造、集中供热管网扩建一期、东平郏路城区段改造等工程完工。湛河综合治理工程取得阶段性成果。城乡一体化示范区完成投资61亿元、增长26%，8条道路具备通车条件，建业十八城、恒大御景半岛等项目进展顺利。舞钢市获批为国家级节约集约模范县（市），郏县创建为国家卫生县城、中国长寿之乡，宝丰县被确定为国家级说唱文化生态保护实验区，鲁山县被授予中国温泉之乡。31个中心镇完成基础设施投资1.3亿元，1813个行政村完成环境综合整治，4个村入选中国传统村落名录，5个村入选全省最美乡村建筑（群）。

（五）发展壮大现代农业，“三农”工作稳步提升。新建高标准粮田14.2万亩，建成玉米饲料粮基地30万亩、生态粮基地43万亩，粮食总产159.8万吨。大力推进现代生态循环农业试验区建设，被确定为省生态循环畜牧业示范市、现代生态循环农业试验区和饲料粮基地建设试点市，被省政府推荐为国家农业可持续发展试验示范区创建重点单位。获100万头生猪冷鲜肉品入沪许可证。与江南大学、河南城建学院共建健康食品协同创新和成果转移中心，推进健康食品产业园建设。新认定“鹰城名优”农业企业5家、农产品7种，新增无公害农产品9个、绿色食品8个，新增省级农业产业化集群3家、省级龙头企业4家、农民合作社319家、家庭农场110个。建成全省第一家市级现代烟草农业科技园，烟叶质量首次位居全省第一。新增农机总动力10.8万千瓦，秸秆“三化”利用率达95%以上，秸秆禁烧工作成效明显。北汝河综合治理、病险水库除险加固等项目进展顺利，涉河违法整治成效明显。新建农村公路540公里，三年行动计划全面完成。农村土地承包经营权确权登记颁证工作有序推进，新增土地流转面积4万亩。

（六）倾心尽力为民惠民，社会大局和谐稳定。十项重点民生工程持续实施，城乡居民人均可支配收入分别增长5.9%和7.6%。强力推进脱贫攻坚，超额完成省定脱贫任务，叶县资金互助扶贫模式在全国推广，鲁山县创建为国家电子商务进农村示范县。城镇新增就业7.8万人，农村劳动力新增转移就业3.8万人。企业退休职工养老金人均月增172元，城乡居民养老保险参保率达到98.6%，机关事业单位养老保险改革稳步推进。城乡低保标准、农村五保供养水平进一步提高，医疗救助制度、临时救助制度不断完善，舞钢市被列入全国救急难试点。新建、改扩建幼儿园45所；超额完成省定“全面改薄”工作任务，新华区、城乡一体化示范区通过国家义务教育发展基本均衡县评估认定；高考本科上线率实现七连增；平顶山学院被确定为省示范性应用技术类型本科院校。新建保障性住房1.3万套，分配公租房3961套。新增200台公交车。建立分级诊疗新模式，基层医疗服务能力持续强化，全面实施两孩政策，人均基本公共卫生服务经费补助标准提高到45元，市精神病医院、市第二人民医院综合楼主体完工。文化惠民活动深入开展，12部戏剧入选省中原文化大舞台剧目库。严格落实安全生产责任制，深入推进隐患排查治理和防控体系建设，安全生产形势持续稳定。信访稳定工作成效明显，社会治安综合治理不断强化，食品药品监管得到加强，处置非法集资工作有序开展，社会大局保持和谐稳定。

一年来，我们自觉接受人大法律监督和工作监督、政协民主监督、社会监督，认真办理人大代表建议和政协委员提案，支持民主党派、工商联和无党派人士参政议政。坚持依法行政，加强“七五”普法宣传教育，完善重大行政决策制度，法治政府建设取得新进展。着力强化“四个意识”，落实全面从严治党主体责任，深入开展“两学一做”学习教育，干部作风进一步好转。加强国防教育、国防后备力量及人民防空建设，驻平解放军、武警部队和民兵预备役人员为地方发展作出新贡献，实现全国双拥模范城“五连冠”。妇女儿童、残疾人、红十字、慈善等事业健康发展。民族宗教、机关事务、审计、统计、史志、档案、地震、气象等工作取得新成绩。

各位代表!

惟其艰难，方显勇毅；惟其磨砺，始得玉成。在严峻复杂的形势下,作为资源型城市能够取得这样的成绩，实属不易。这是省委、省政府和市委正确领导的结果，是市人大、市政协大力支持的结果，是全市人民共同奋斗的结果。在此，我代表市人民政府，向广大工人、农民、知识分子、干部，向驻平人民解放军指战员、武警官兵、公安民警及社会各界人士，致以崇高敬意！向关心支持平顶山发展的海内外友好人士，表示诚挚感谢！

在肯定成绩的同时，我们也清醒地认识到，受经济下行大势影响，主要经济指标增速较低，个别指标未能达到年初预期；产业结构依然偏重，经济增长后劲不足，具有当期拉动和长期支撑能力的大项目较少。城市建设与管理同先进地区相比还有差距，城市综合承载能力需要进一步提高。环境污染治理任务仍很艰巨，非法集资、问题楼盘、安全生产等领域风险隐患不容忽视。政府职能转变还不到位，一些工作人员不会为、不想为、不敢为、乱作为问题依然存在。对此，我们一定勇于担当、努力工作，认真加以解决，决不辜负全市人民的期望！

二、2017年工作安排

今年是党的十九大召开之年、供给侧结构性改革的深化之年，也是落实省、市党代会精神的开局之年和建市60周年。总的看，我市面临的发展环境依然错综复杂。一方面，世界经济增长乏力，全球贸易持续低迷，国内经济“三期叠加”，下行压力依然较大。我市自身结构性矛盾突出，统筹稳增长与调结构、促转型任务更加艰巨。另一方面，经过多年发展，我市产业结构逐步优化、综合实力持续提升，当前既有国家政策的支持，又有承接产业转移的机遇，更有自身优越的资源禀赋、坚实的产业基础、日趋完备的载体平台。特别是随着国家部署在我省的一系列战略深入实施，将为我们带来政策、资金、技术、项目等方面的诸多利好；随着郑万铁路、沙河复航、鲁山机场等项目的建设，将有效改善我市综合交通条件，有助于发展临铁经济、临港经济、临空经济。只要我们坚定信心、鼓足干劲，迎难而上、奋力拼搏，就一定能够牢牢掌握发展主动权，开创转型发展新局面。

今年政府工作的总体要求是：全面贯彻党的十八大和十八届三中、四中、五中、六中全会精神，更加紧密地团结在以习近平同志为核心的党中央周围，深入贯彻习近平总书记系列重要讲话精神和治国理政新理念新思想新战略，统筹推进“五位一体”总体布局和协调推进“四个全面”战略布局，认真落实中央、省委、市委经济工作会议和省十次党代会、市九次党代会各项部署，适应把握引领经济发展新常态，牢固树立和贯彻落实新发展理念，坚持以稳中求进为工作总基调，坚持以供给侧结构性改革为主线，坚持以转型发展为主题，发挥优势打好“四张牌”，聚焦“1461”发展思路，以三年行动计划为抓手，持续提高发展质量和效益，坚决守住安全、民生、生态三条底线，全面做好稳增长、促改革、调结构、惠民生、防风险各项工作，在决胜全面小康征程中迈出坚实步伐，以优异成绩迎接党的十九大胜利召开。

主要预期目标是:在结构优化、活力增强、环境改善的基础上，生产总值增长7.5%，一般公共预算收入增长5%，固定资产投资增长12%，社会消费品零售总额增长11%，进出口总值稳定增长，城镇新增就业6.4万人，常住人口城镇化率提高1.4个百分点，居民收入与经济增长基本同步，节能减排、环境保护等约束性指标完成省下达任务，结构性改革取得实质性进展。

各位代表！

落实总体要求、做好今年政府工作，最重要的是切实增强“四个意识”特别是核心意识、看齐意识，坚决维护以习近平同志为核心的党中央权威，用新发展理念指导实践、推动工作。最关键的是坚定不移地贯彻省委、省政府和市委决策部署，切实做到“一坚持、三突出、五着力”，妥善处理好“四个关系”，全力打好四大攻坚战，把市九次党代会提出的发展思路落实到具体工作中，将决胜全面小康、建设资源型城市可持续发展示范区的美好蓝图变为现实。最根本的是落实以人民为中心的发展思想，把人民放在最高位置，一切为了人民、一切依靠人民，始终为人民利益而奋斗。

实现上述目标，必须重点抓好以下八个方面工作：

（一）激发市场需求潜力，促进经济稳定增长。坚持供给结构与需求结构有效匹配、消费升级和有效投资良性互动，积极调整需求结构，持续释放需求潜力。

推动消费升级。加快完善市场体系规划布局，以工业消费品和食品为重点，增加高质量、高水平产品的有效供给，扩大产品消费。加快发展健康护理、体育健身、绿色消费等新型消费业态，扩大服务消费。多渠道增加居民特别是中低收入群体收入，提高居民消费能力。加快现代流通体系、冷链物流、公共信用信息平台等消费基础设施建设，持续开展消费专项整治行动, 促进消费结构升级。

扩大有效投资。以“33626”投资提速工程为抓手，完善市级领导联系重点项目等制度，持续开展重大项目集中开工活动，加大项目建设力度，实施重点项目352个，完成投资600亿元以上。围绕能源、交通、水利、环保、农业、林业、重大市政工程等七大领域，精心谋划项目、包装项目，建立有效的工作推进机制，狠抓PPP项目三年行动计划实施，激发社会资本投资活力。

振兴实体经济。深化企业大服务专项行动，实施“百千万”助力企业计划，构建企业服务长效机制。深入推进政银企合作，加强对小微企业和劳动密集型企业的金融服务，支持企业上市直接融资，加大债券市场融资力度。创新土地供应机制，探索工业用地先租后让、弹性年期出让等供应方式，做好耕地占补平衡工作，为项目建设提供用地保障。深入推进全民技能振兴和职教攻坚工程，强化人力资源保障能力。

防范化解风险。严格地方政府性债务管理。引导企业强化风险意识，合理控制资产负债率，鼓励金融机构帮助暂时遇到困难的企业渡过难关。加强不良资产、债券违约、企业互保等领域风险防控，持续做好防范、打击和处置非法集资工作，坚决守住不发生区域性系统性风险的底线。

（二）加快推动产业转型，提升供给质量效率。加大供给侧结构性改革力度，积极创建国家老工业基地和资源型城市产业转型升级示范区，培育壮大经济发展新动能。

深入推进“三去一降一补”。认真落实推进供给侧结构性改革政策措施，巩固拓展既有成效。去产能方面，以煤炭行业为重点，合理安排产能退出时序和进度，完善矿井退出、职工分流安置、资产债务处置等机制，完成省定煤炭产能退出任务。加强监管，防止已化解过剩产能死灰复燃。去库存方面，完善购房信贷政策，扩大公积金受益覆盖面，加大农民购房支持力度，提高棚改货币化安置比例，优化商品房供给结构，抓好问题楼盘处置，促进房地产市场平稳健康发展。去杠杆方面，把降低企业杠杆率作为重点，推动企业加快兼并重组、债务重组，盘活存量资产，推进债权转股权，发展股权融资，优化负债结构，逐步降低杠杆率。降成本方面，落实好降成本政策措施，着力降低企业生产要素和制度交易成本，引导企业加强内部管理，堵塞跑冒滴漏，实现降本增效。补短板方面，结合“十三五”发展规划，在产业转型、脱贫攻坚、生态环境、基础设施、公共服务、关键共性技术等薄弱环节实施一批重大项目，补齐发展短板。

持续优化工业结构。大力实施产业转型升级行动计划，坚持做大总量和调优结构并重、改造提升传统产业和培育战略性新兴产业并举，设立首批50亿元的产业升级引导基金，着力打造中国尼龙城、中部能源城、中原电气城、全国重要的不锈钢产业基地。着力建设中国尼龙城。以延链补链强链为重点，深度推进与国内外化工龙头企业战略合作，抓好尼龙新材料产业园建设，加快推进20万吨己内酰胺、15万吨环己酮、5万吨尼龙6帘子布等项目。支持平煤神马集团与科研院所组建尼龙高端注塑等国家级实验室，研发尼龙新材料产业关键技术，开发高端产品。着力建设中部能源城。大力调整煤炭产品结构，增加主焦煤产量，积极发展氢、苯、焦油等深加工产品，以京宝焦化、中鸿焦化、洁石焦化为依托，打造煤焦化产业集群。大力发展新能源、可再生能源和储能产业，加快推进中广核叶县4.8万千瓦风电、中民新能40万千瓦光伏发电、鲁山花园沟抽水蓄能电站等39个重大项目。加快建立新能源汽车产业研究院和产业联盟，发展壮大新能源整车和零部件生产企业。注重发展氢能源产业。着力建设中原电气城。依托高压电器装备技术研发和品牌优势，突出龙头带动、集群配套，加快推进平高电气高端装备制造产业园等22个重大项目。着力建设不锈钢产业基地。依托宝丰不锈钢产业园和舞钢公司优势，大力发展不锈钢和特种钢板高端产品，加快推进翔隆不锈钢产业园等13个重大项目，打造全国重要的不锈钢加工中心、交易中心和配送中心。着力建设特色产业集群。实施产业集聚区“一区一主业”集群发展计划，培育高端装备、光电信息、机械装备、摩托机车及零部件等特色产业集群，产业集聚区完成投资580亿元，一星级产业集聚区达到5个以上。

积极推动服务业发展。全面实施服务业发展行动计划，提高服务业发展规模和水平，第三产业增长9%以上。加强载体和项目建设。健全服务业“两区”工作机构和推进机制，加快推进鹰城中心、华耀城等项目，启动商品直销中心项目建设，争取3家特色商业区实现晋位升级。加快神前陶瓷专业园、圣光医药物流园、南部快递物流园等专业园区建设，力争成功申报一批省级专业园区。设立服务业发展专项基金，引导社会资本投向服务业领域。发展全域旅游。编制全域旅游发展规划，实施规划引领、品牌提升、乡村旅游提质、形象推广等工程，加快推进海内外知名的旅游目的地建设。发挥“山、佛、汤、寺、衙、湖”等资源优势和汝瓷、花瓷、唐钧、金镶玉等产品优势，推进4个旅游主体功能区和1条旅游生态廊道建设，加快白龟湖国家湿地公园、大香山文化旅游区、尧山景区环路和停车场、舞钢龙凤湖旅游度假区等项目建设，支持舞钢、鲁山创建国家全域旅游示范区，支持叶县开展农业公园建设试点，推进开发一批特色旅游片区、创建一批全域旅游示范乡镇。

培育网络经济。以智能制造为主攻方向，推进制造业与互联网深度融合，助力制造业转型。深入实施“互联网+”行动计划，加快互联网在制造业、服务业、能源等领域应用，推动互联网医疗、互联网教育、互联网体育等业态快速发展。积极引进和培育龙头电商，全面推进电子商务进农村、进社区、进企业。建设平顶山大数据云计算中心，推动智慧城市建设取得实质性进展。完善网络基础设施，实现市县乡有线宽带和无线网络全覆盖。以“文化创意+”为引领，大力发展休闲娱乐、工艺美术等创意产业，加快培育创意园区、企业和品牌。

提升产业素质。深入推进质量强市建设，加快平顶山制造向平顶山创造转变、平顶山产品向平顶山品牌转变。大力实施标准化引领工程，引导企业对标一流企业，加强全流程管理，提升创新能力，加快中高端产品开发。探索“政府引导+市场化运作”模式，开展知名品牌示范区创建，提升产业集群发展水平。弘扬企业家精神和工匠精神，加大企业家培训力度，以企业家素质提升引领产业素质提升。

（三）做强做优现代农业，破解“三农”发展难题。以创建国家农业可持续发展试验示范区为抓手，全面落实“循环农业+品牌农业+协同农业”新战略，推进农业供给侧结构性改革。调整农业结构。统筹推进粮食“双高”创建、生态粮饲料粮基地、农业产业化集群、都市生态农业建设，突出抓好健康食品产业园与生猪、大豆、鸡和蛋品深加工等“1+3”重点产业，推动农林牧深度融合、种养加一体、产供销相衔接。全年粮食总产稳定在150万吨以上。大力发展品牌农业，着力打造优质农业产品，拓展上海等大城市目标市场。增加农民收入。健全农业社会化服务体系，开展“互联网+现代农业”行动，提高农产品加工转化率，增加经营性收入。探索“资源变资产、资金变股金、农民变股东”模式，盘活资产，增加财产性收入。落实惠农补贴政策，增加转移性收入。加强技能培训，提高转移就业能力，增加工资性收入。加大农业保险力度，帮助农民避灾减损。深化农村改革。全面完成土地承包经营权确权登记颁证，开展房地一体的农村权籍调查，颁发统一的不动产权证书。落实承包土地所有权、承包权、经营权分置政策，支持宝丰县农村承包土地经营权抵押贷款试点工作。抓好农村集体经济组织股份合作制改革试点工作。深化林业、水利、供销社等改革。全面完成农业普查任务。改善农村条件。继续开展农村人居环境整治。加快交通、供水、供电、信息、污水垃圾处理等农村基础设施建设，推进基本公共服务和社会服务向农村覆盖。抓好北汝河综合开发治理、石漫滩水库等除险加固及防洪建设。实施农村饮水安全巩固提升工程，加强农村危房改造，新改建农村公路480公里。

（四）科学推进新型城镇化，推动城乡区域协调发展。以百城建设提质工程为抓手，以“三改三拆”为突破点（工矿区改造、老旧居民区改造、城中村改造，违法建筑拆除、断头路用地拆迁、重点项目用地拆迁），推动城镇化生态、融合、适度、集约、高效发展。优化城镇布局。积极融入中原城市群，协同推动西部转型创新发展示范区建设。启动城市总体规划修编，完成全域规划编制，全面开展城市设计，推动宝丰、叶县、鲁山、石龙区与中心城市协同联动发展，增强舞钢、郏县支撑地区发展能力，加快构建“一核三组两星”的城镇化格局。提升城市综合承载能力。规划实施白龟湖环湖区域综合治理及赛道建设、沙河全面治理、高铁片区核心区、市区南部快速通道、大西环和大南环、北部矿区生态建设、城市轨道交通规划建设、高速路出入口全面整治、鲁山机场、打通城市断头路和城市综合管廊建设等十大工程，打造内畅外联、生态环保的城市环境。加快南水北调焦庄水厂及配套管网建设。加强燃气、供热设施和管网建设改造，推进东线热网改造和湛河以南供热管网建设。城乡一体化示范区实施项目155个、完成投资71亿元，推进应河治理、体育公园、市第一人民医院新院区、市中医医院新城区分院等项目，确保市一中新区学校（一期）投入使用。抓住棚改三年行动计划的政策机遇，强力推进城区棚户区改造，全面完成棚改任务。开展生态修复和城市修补，推动海绵城市建设，启动申报“中国人居环境奖”。加快推动引水、供水、四库一渠联动等工程，构建多源互补、调控自如的全域生态水网体系。推进湛河综合治理，抓好18条支沟整治、沿岸绿化美化和生态建设。持续开展创卫“五个一”竞赛，迎接国家卫生城市、国家园林城市届满复审。完成城市综合执法体制改革，发挥好数字化城管作用，提升城市精细化管理水平。加快构建综合交通体系。推进郑万铁路、郑西高速和周南高速平顶山段、沙河复航、平叶快速通道等项目建设，做好呼南高铁、林唐高速、焦平高速平顶山段、平鲁快速通道、南北绕城高速等项目前期工作。大力发展公交事业，加强港湾及场站建设。加快县城和小城镇建设。突出高水平编制规划、完善城市功能、推进产城融合、提高人居环境质量等关键环节，上半年所有县（市）完成城乡总体规划编制和城市设计，年底前完成市政公用设施和公共服务设施专项规划编制，谋划推进一批水电路气暖等基础设施项目和公共服务项目，实施鲁山、舞钢、叶县等污水处理改扩建工程。加大对县（市）支持力度，提升县级投融资平台的融资能力。规划建设一批富有活力和魅力的特色小镇，改造建设一般的小城镇。促进农业转移人口市民化。坚持“一基本两牵动三保障”，加快户籍制度改革和居住证制度“双落地”，推动城镇基本公共服务常住人口全覆盖；用好城镇建设用地与农业转移人口落户数量挂钩政策，建立农业转移人口市民化激励机制。

（五）持续强化开放带动，拓展经济发展空间。坚持上下联动、协同发力，推进更深层次更高水平的对外开放。扩大开放招商。坚持把招商引资作为一号工程，围绕“十三五”规划和“三城两地一区”发展目标，瞄准重点区域、对接重点客商，实施精准招商、平台招商，持续开展专题招商、产业链招商、以商招商和亲情招商。加强跟踪服务和督促检查，全力推动已签约项目落地。大力推进现代服务业、现代农业、社会事业、城乡建设等领域开放，努力在金融保险、商贸物流、文化旅游等方面取得新突破。拓展对外贸易。深度融入国家“一带一路”战略，推动能源、化工、装备制造等行业的优势企业到境外开拓国际市场，开展工程承包及经济技术合作。巩固神马、舞钢、平高、平棉等知名品牌，抓好隆鑫机车、力帆车业、真源制衣、鲁吉兰等企业出口，持续推动出口基地建设。加强与国内外知名电子商务平台合作，加快跨境电子商务发展。优化服务环境。全面推行首问负责、服务承诺、一次性告知、限时办结、责任追究等制度，持续提升行政服务质量和水平。注重招商与亲商、安商有机结合，完善招商引资优惠政策，保护投资者合法权益。加快建设郑州海关和河南出入境检验检疫局平顶山办事处。

（六）深入推进改革创新，增强发展内生动力。围绕破解经济社会深层次矛盾和问题，加快推进基础性关键性改革，全面推进创新驱动发展，提升区域竞争优势。

以改革破解发展瓶颈。深化国企改革攻坚。大力支持平煤神马集团、平高集团、舞钢公司等大型国有企业发展。继续把剥离企业办社会职能、完善法人治理结构、深化内部改革作为国企改革的重点，积极稳妥接收驻平央企、省属企业分离移交的办社会职能，启动市属国有企业剥离办社会职能工作。支持驻平央企、省属企业进行混合所有制改革。深化财税金融体制改革。提高县级基本财力保障能力，强化绩效导向，优化支出结构，深化财政资金投入机制改革；推进税收征管体制改革，完善社会保险费征管新机制。积极引进股份制银行，鼓励新设村镇银行，全面完成农信社组建农商行工作。深化投融资体制改革。加快政府投融资公司转型发展，规范国有资本投资、运营公司管理，整合重组投融资公司功能，做大做强市发展投资公司、市财信投资公司等六大投融资公司。坚持改拨为投、投贷结合，发挥好科技创新及先进制造业、农业发展等基金作用。加大土地收储力度，盘活用好土地资源，实现土地收储、开发、出让的良性循环和滚动发展。统筹推进医疗、医保、医药联动改革。全面推进城市公立医院综合改革，深化县域综合医改，推进分级诊疗制度建设，开展城乡卫生基础设施建设规范提升工程，推广家庭医生签约服务；健全基本医保稳定可持续筹资和报销比例调整机制，推行按病种付费为主、多种付费方式相结合的医保支付方式改革；深化药品供应保障制度改革，落实国务院关于所有公立医院取消药品加成的要求。同时，加快电力体制改革，推进盐业、价格、文化体制等改革。

以创新释放发展活力。加快推进以科技创新为引领的全面创新，以创新促发展求突破。培育壮大创新主体。加强政策引导支持，整合现有国家级、省级科技企业孵化器功能，吸引国内外高层次人才及团队带技术、带成果、带项目到我市创新创业和转化成果，大力培育创新型领军企业和科技型中小企业，力争孵化科技型小微企业100家以上，转化先进适用高新技术成果100项以上。开展国家知识产权示范市创建。加强创新平台建设。鼓励企业与科研院所、高等院校合作研发或共建创新平台，推进大中型企业省级以上研发机构全覆盖，支持平高集团与清华大学组建电气技术联合研究中心、与平顶山学院共建河南省高压智能电器工程技术研究中心。加大技术研发力度。建立健全科技金融服务体系，引导社会资本投资科技创新。瞄准我市产业转型升级的核心技术、关键技术，实施高压直流断路器、高性能聚酰胺66工业丝产业化等重大科技专项，加快开发具有较强市场竞争力的新产品。激发创新创业活力。坚持创新创业结合、线上线下结合、孵化转化结合，推动商业模式创新和业态创新，厚植双创良好生态，营造良好创业氛围。

以服务繁荣非公经济。落实促进非公有制经济发展若干意见，健全产权保护机制，鼓励建立现代企业制度，着力解决融资难、融资贵等问题，大力发展非公企业。着力营造“亲”“清”政商关系，关心爱护企业家，支持企业家创新创业。加快商事制度改革，探索试行“多证合一”，推进登记注册全程电子化，推动“双随机、一公开”监管全覆盖，开展减证便民专项行动。

（七）打好环境污染防治攻坚战，提高绿色发展水平。牢固树立绿水青山就是金山银山的理念，全面推进生态文明建设，努力建设天蓝、地绿、水净的美丽鹰城。加强环境整治。继续开展大气污染防治攻坚战，聚焦重点区域、重点时段，实施预算式管理，推进全民治污、依法治污、科学治污，完成空气质量改善目标任务，确保PM10降到110微克/立方米，PM2.5降到67微克/立方米，空气优良天数达到203天。打响水污染治理攻坚战，落实“河长制”，一河一策、精准治污，系统推进水污染防治、水生态保护和水资源管理，确保集中饮用水源地水质达标率保持100%、出境断面水质综合达标率完成省定目标。启动土壤污染状况调查，加强农业面源污染治理、重金属污染防治和土地复垦工作。加强生态建设。深入实施林业生态建设提升工程，围绕水网、田网、路网“三网合一”，生态林、产业林、景观林“三林共建”，抓好高速、国道、省道、大中型河流、南水北调等生态廊道建设，启动北部山区绿化工程规划建设，积极争取国家储备林基地建设项目，完成造林28.4万亩。加强节能减排。建立能源、水资源消耗总量和强度目标预警制度，组织开展重点行业能效提升工程。建立完善控制污染物排放许可制度，实施重点行业温控行动。大力发展循环经济，推进大宗固废综合利用和园区循环化改造。加大节能环保技术、工艺和装备研发力度，促进节能环保产业加快发展。健全环保机制。完善“党政同责、一岗双责”的领导机制和党委领导、政府负责、环保牵头、部门协作、齐抓共管的环保运行机制，健全环境保护督察、损害赔偿、责任追究制度，确保环保政策措施落到实处。

（八）切实保障改善民生，促进社会和谐稳定。加大财政对民生的投入，集中力量办好关系群众切身利益的15件民生实事，进一步增强人民群众获得感。

深入推进脱贫攻坚。坚持精准扶贫、精准脱贫基本方略，强化领导、落实政策，创新机制、加大投入，在激发内生动力、改善发展条件、提升扶贫实效、促进均衡发展上下功夫，大力实施产业扶贫，加强农村低保与扶贫政策有效衔接，统筹做好非贫困县贫困村和非贫困村贫困户脱贫工作，确保8万贫困人口实现脱贫、叶县脱贫摘帽。

稳定扩大社会就业。突出做好高校毕业生、农村转移劳动力、城镇困难人员、退役军人等就业工作，妥善解决化解产能过剩企业分流职工再就业问题，支持大学生和返乡农民工创业。

提升社会保障水平。全面实施全民参保登记计划，进一步完善城乡居民基本养老保险制度，落实企业年金和职业年金基金管理办法。深化机关事业单位养老保险制度改革，落实机关公务员职务与职级并行制度。实行统一的城乡居民基本医保制度。落实困难残疾人生活补贴和重度残疾人护理补贴制度。完善失业保险制度。推进保障性安居工程建设，新建保障性住房5604套，续建项目基本建成6000套，完成公租房分配2385套。大力发展慈善事业。完善社会养老服务体系，推动老年教育发展。健全社会救助制度，加强农村“三留守”人员关爱服务和困境儿童保障，适当提高城乡居民最低生活保障和特困人员供养救助标准。

全面发展社会事业。启动第三期学前教育三年行动计划。持续实施“全面改薄”和扩充城镇义务教育资源五年计划，做好义务教育发展基本均衡县创建工作，完善区域内经费保障一体化机制和乡村教师激励政策。整合职业教育资源，组建平顶山职业技术学院。支持平顶山学院、河南城建学院、平顶山工业职业技术学院、河南质量工程职业学院等高校发展。积极创建全国文明城市，推进核心价值观示范点、主题游园和“鹰城好人馆”建设，深化行业道德实践和群众性精神文明创建。抓好公共文化服务体系示范区创建，办好中国•河南第二届坠子大会，积极创建中国曲艺名城，加快市科技馆、档案馆、党史馆、地方志馆和老年活动中心等项目建设。持续实施基层卫生人才工程，引导医疗卫生工作重心下移、资源下沉，深化计生服务管理改革，加强重大疾病防控，提升基本公共卫生服务均等化水平，推进健康鹰城建设。巩固全民健身示范城市创建成果，办好市第四届全民健身大会，建设市体育运动学校综合训练馆。

推进社会治理创新。严格落实各类监管主体安全生产责任制，深化重点行业、重点领域专项整治，排查消除各类事故隐患，坚决遏制重特大事故发生。完善信访工作机制，及时预防化解各类群体性事件。高度重视食品药品安全监管，坚决把好人民群众饮食用药安全的每一道关口。深化平安鹰城建设，加快构建立体化信息化社会治安防控体系，提高基层社会治理能力，完善反恐和打防犯罪机制，依法惩治盗窃、抢劫、电信网络诈骗、侵犯个人信息等多发性犯罪和黑恶势力犯罪，确保社会和谐稳定、人民安居乐业。

落实国家国防和军队改革的有关政策措施，加强国防教育和国防后备力量建设,支持驻平解放军、武警部队、预备役部队、民兵和人民防空建设，争创省级双拥模范城“六连冠”。全面实施“七五”普法规划，深入开展法治鹰城建设。促进民族团结、宗教和睦。支持工会、共青团、妇联等人民团体发挥桥梁纽带作用。继续做好对台、统计、气象、地震、国家安全等工作。

三、全面加强政府自身建设

决胜全面小康、加快转型发展必须加强政府自身建设，提升新常态下推动发展的能力水平。

（一）强化“四个意识”，坚定政治方向。坚持党的领导，牢固树立政治意识、大局意识、核心意识、看齐意识，始终在思想上政治上行动上与以习近平同志为核心的党中央保持高度一致。严守政治纪律，恪守政治规矩，自觉坚持党的基本路线，坚决贯彻中央大政方针，坚决落实中央、省委、市委决策部署，始终做到政治坚定、对党忠诚。

（二）强化责任担当，转变政府职能。坚持干字当头，真抓实干、埋头苦干、创造性地干，进一步压缩会议、精简文件、减少应酬，不简单以会议贯彻会议、以文件落实文件。深化“放管服”改革，动态调整行政审批事项，全面推行权力、责任和效率清单，清理规范中介服务，加强公共资源交易管理，深入实施模拟审批，推进 “互联网+政务服务”建设。强化督查问责，深化懒政怠政为官不为专项治理，力破“中梗阻”。健全激励和容错纠错免责机制，让干部敢干事、能干事、想干事，形成干事创业的良好氛围。

（三）强化依法行政，建设法治政府。严格执行人大及其常委会的决议决定，自觉接受法律监督和工作监督，主动接受政协民主监督，认真办理人大代表建议和政协委员提案，广泛听取民主党派、工商联、无党派人士意见,自觉接受舆论监督和社会监督。着力推进法治政府建设，把依法行政、依法办事贯穿政府决策、执行、落实的全过程，严格按照法定权限和法定程序行使权力，提高运用法治思维和法治方式深化改革、推动发展、化解矛盾、维护稳定的能力。积极稳妥推进地方立法，依法依规制定规范性文件。

（四）强化为民宗旨，坚持廉洁从政。真诚倾听群众呼声，真实反映群众意愿，真情关心群众疾苦，着力解决群众最关心、最直接、最现实的利益问题，多为群众办实事、解难题、谋福祉。严格遵守党章党规党纪，贯彻落实“八项规定”精神，切实做到“三严三实”，持续纠正治理“四风”问题。加强行政监察和审计监督。落实党风廉政建设主体责任和“一岗双责”，加强惩治和预防腐败体系建设，严格执行廉洁从政各项规定，努力实现干部清正、政府清廉！

各位代表！

风劲潮涌，正可扬帆破浪；任重道远，更须勇攀高峰。让我们更加紧密地团结在以习近平同志为核心的党中央周围，在省委、省政府和市委的坚强领导下，万众一心、锐意进取，不忘初心、继续前进，加快建设资源型城市可持续发展示范区、奋力谱写决胜全面小康新篇章，以优异成绩迎接党的十九大胜利召开！
